# Rationalization of Activity Cliffs of a Sulfonamide Inhibitor of DNA Methyltransferases with Induced-Fit Docking

**DOI:** 10.3390/ijms15023253

**Published:** 2014-02-21

**Authors:** José L. Medina-Franco, Oscar Méndez-Lucio, Jakyung Yoo

**Affiliations:** 1 Mayo Clinic, 13400 East Shea Blvd., Scottsdale, AZ 85259, USA; 2 Unilever Centre for Molecular Science Informatics, Department of Chemistry, University of Cambridge, Lensfield Road, Cambridge CB2 1EW, UK; E-Mail: om268@cam.ac.uk; 3 Life Science Research Institute, Daewoong Pharmaceutical Co., Ltd., 72, Dugye-Ro, Pogok-Eup, Gyeonggi-do 449-814, Korea; E-Mail: jyoo161@daewoong.co.kr

**Keywords:** drug design, enzyme inhibition, epigenetics, induced-fit docking, protein-ligand interactions

## Abstract

Inhibitors of human DNA methyltransferases (DNMT) are of increasing interest to develop novel epi-drugs for the treatment of cancer and other diseases. As the number of compounds with reported DNMT inhibition is increasing, molecular docking is shedding light to elucidate their mechanism of action and further interpret structure–activity relationships. Herein, we present a structure-based rationalization of the activity of SW155246, a distinct sulfonamide compound recently reported as an inhibitor of human DNMT1 obtained from high-throughput screening. We used flexible and induce-fit docking to develop a binding model of SW155246 with a crystallographic structure of human DNMT1. Results were in excellent agreement with experimental information providing a three-dimensional structural interpretation of ‘activity cliffs’, e.g., analogues of SW155246 with a high structural similarity to the sulfonamide compound, but with no activity in the enzymatic assay.

## 1. Introduction

**Figure 1.**
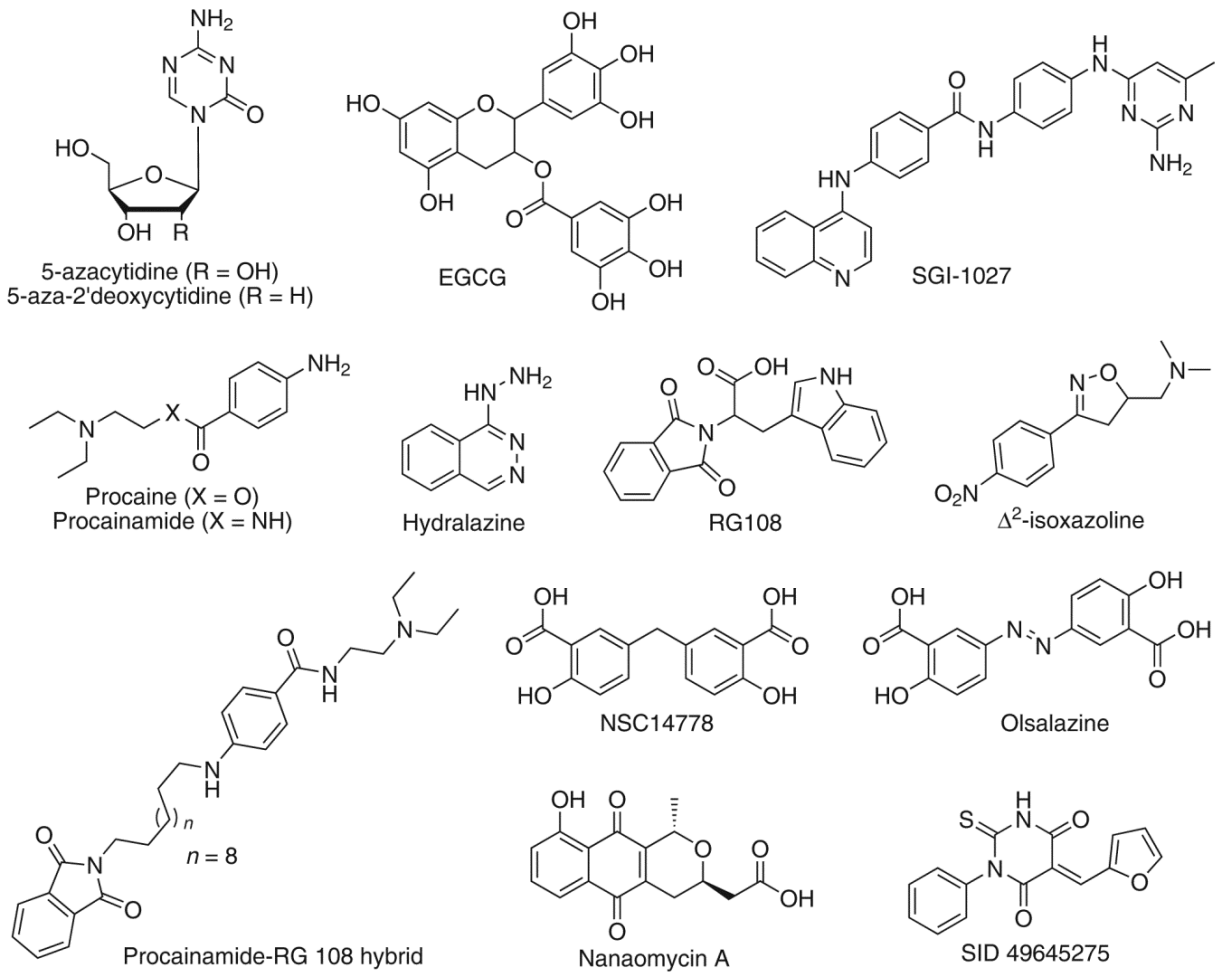
Selected compounds associated with DNA methyltransferases (DNMT) inhibition and hypomethylating agents.

DNA methyltransferases (DNMTs) are a family of enzymes that catalyze the transfer of a methyl group from *S*-adenosyl-l-methionine (SAM) to the carbon-5 position of cytosine residues, leading to an epigenetic modification [1]. As part of the mechanism of methylation, *S*-adenosyl-l-homocysteine (SAH) is generated. Three active forms of DNMT have been identified in mammals: DNMT1, DNMT3A/3B, and DNMT3L. DNMT1, which is the most abundant of the three, is involved in the maintenance of methylation patterns, whereas DNMT3A and DNMT3B are responsible for *de novo* methylation [2]. DNMT3L is required for the catalytic activity of DNMT3A and DNMT3B, though it lacks catalytic activity [3]. These enzymes regulate gene expression. In particular, DNMT1 is responsible for duplicating patterns of DNA methylation during replication and is essential for mammalian development and cancer cell growth [4]. Since inappropriate methylation is thought to be a key antecedent step in transformation [5], it is anticipated that DNA hypomethylating drugs that act on DNMTs may be effective anti-cancer agents. DNMT inhibitors are also promising new drugs for the treatment of brain disorders [6]. There have been rapid synthetic approaches based on the conjugation of known inhibitors such as procainamide-RG108 hybrid (Figure 1). Procainamide is a potential DNMT inhibitor approved by the FDA as antiarrhythmic, and RG-108 was identified via virtual screening (Figure 1). Currently, 5-azacytidine and 5-aza-2′-deoxycytidine are the only two DNMT inhibitors clinically in use for the treatment of certain types of cancer [7]. Since there are concerns about the low specificity and clinical toxicity of these nucleoside analogues [7] it is convenient to identify novel non-nucleoside DNMT inhibitors. Compounds with different chemical classes are associated with demethylating activity, and some of them were proposed as DNMT inhibitors (Figure 1). Most of these compounds were identified fortuitously. Therefore, there is an increased interest to identify novel DNMT inhibitors using systematic computational and experimental screening of chemical databases. For example, our group identified NSC 14778 (Figure 1) and other DNMTs with distinct chemical scaffolds using virtual screening followed by experimental validation [8]. NSC 14778 was the starting point to identify olsalazine as a novel hypomethylating agent using a computer-guided drug repurposing strategy [9]. The increased availability of crystallographic structures of DNMTs have boosted the use of molecular docking and other structure-based computational approaches to suggest hypothesis of the binding mode of DNMT inhibitors [10,11].

**Figure 2.**
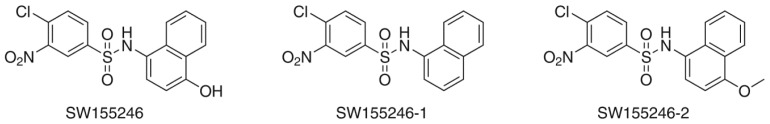
Chemical structures of **SW155246** and structural analogues studied in this work.

Experimental high-throughput screening (HTS) is starting to be used as an approach to identify novel inhibitors of DNMTs [12]. A recent HTS used a scintillation proximity assay to evaluate ~180,000 molecules; the hit confirmation rate was low (0.03%) and most of the hits were found to be active due to the generation of reactive oxygen species. Only **SW155246** (Figure 2) showed human DNMT1 activity (IC_50_ = 1.2 μM) without affecting protein levels or generating reactive oxygen species [13]. A focused structure-activity relationship (SAR) analysis showed that the hydroxyl group of **SW155246** was essential for its activity; loss of the hydroxyl group (**SW155246-1**) or addition of a methylated oxygen on the 1-position of the naphthyl ring (**SW155246-2**) (Figure 2) completely abolished the ability of this compound to inhibit human DNMT1 activity in vitro and reduced the cell-based cytotoxicity [13]. This is an example of an ‘activity cliff” [14,15], *i.e.*, a small change in the structure dramatically affects the biological activity. However, the binding mode of **SW155246** with DNMT1 and the corresponding rationalization of such activity cliffs have not been reported.

In this work, we elucidate the binding mode of **SW155246** with human DNMT1 providing a structure-based interpretation of the observed SAR *i.e.*, loss of activity upon removal or methylation of the hydroxyl group. For this purpose, we used molecular docking with a crystallographic structure of human DNMT1 recently published. In order to account for protein flexibility, we conducted induced-fit docking (IFD). We have previously used IFD to model other DNMT inhibitors [16]. The binding mode of **SW155246** was compared to the observed binding mode of the potent co-crystal inhibitor sinefungin. Of note, **SW155246** is a sulfonamide compound and related sulfonamides have shown important bioactivities [17]. We anticipate that the outcome of this work may help to design novel sulfonamides as inhibitors of DNMTs.

## 2. Results and Discussion

Docking of **SW155246** and their structural analogues with human DNMT1 was preceded by validation of the docking approach.

### 2.1. Validation of the Docking Protocol

**Figure 3.**
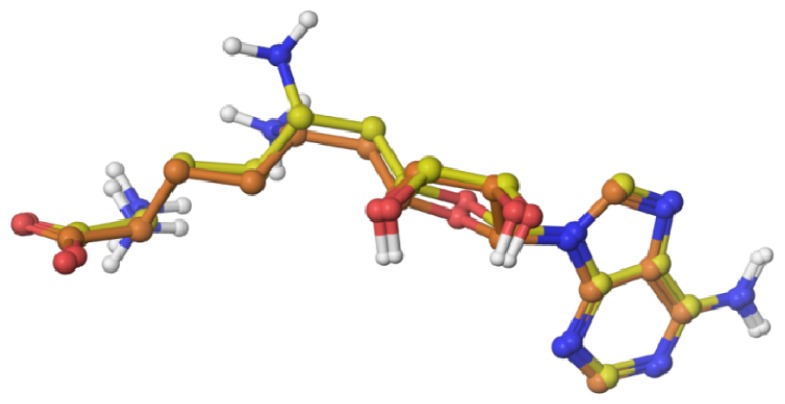
Validation of the docking protocol: comparison of the co-crystal (yellow) *vs*. predicted (orange) binding mode of sinefungin with Glide XP.

In order to validate the docking protocol, we re-dock the co-crystal inhibitor sinefungin with the crystal structure of human DNMT1 (PDB ID: 3SWR). The docking protocol, described in the Experimental Section, successfully reproduced the bound conformation of sinefungin with a root-mean square deviation (RMSD) of 0.547 Å. Figure 3 shows a comparison of the predicted binding mode with Glide XP with the bound position observed in the crystallographic structure. Results were in agreement with previous reports that showed the capability of Glide XP to reproduce the co-crystal position of sinefungin [16].

### 2.2. Flexible Docking

First we did regular docking of the compounds in Figure 2 considering the ligands as flexible but retaining the protein as a rigid structure. The rank-order of the Glide XP docking scores of **SW155246** and structural analogues with human DNMT1 were consistent with the reported activity. **SW155246** showed the lowest, *i.e.*, most favorable Glide XP docking score (−4.817 kcal/mol) as compared to the analogues **SW155246-1** (−4.312 kcal/mol) and **SW155246-2** (−4.370 kcal/mol) (Table 1). However, as discussed in the next section, the magnitude of the docking scores and predicted binding modes for all three molecules were different from the results obtained with IFD, in particular for the compounds with the hydroxyl and methoxy groups.

### 2.3. Induced-Fit Docking

It is well-known that protein flexibility plays an important role in the protein–ligand recognition process. For DNMT inhibitors, we previously showed that IFD may have a significant impact on the predicted binding modes and docking scores [16]. To consider protein flexibility we carried out IFD with the three compounds in Figure 2. An alternative approach to address protein flexibility is to conduct docking with different structures available for human DNMT1. In this work, we focused on IFD as this approach provided an excellent agreement with experimental data. Table 1 summarizes the Glide XP and IFDScores obtained with the IFD protocol detailed in the Experimental Section. This table clearly shows that for all three compounds, the Glide XP scores computed with IFD were more favorable than the scores obtained with regular docking where only the ligands are considered flexible but the protein is kept fixed during docking. Similar results were obtained for other DNMT inhibitors [16]. These results further highlight the importance of considering the flexibility of the protein. Although the difference in Glide XP scores between **SW155246** and the two analogues was small, the active compound showed the lowest score in agreement with the experimental data. However, despite the fact that significant progress has been made to improve scoring functions, it is well-known that the docking scores still have a large room from improvement [18]. Therefore we focused the analysis of the binding modes predicted with IFD.

**Figure 4.**
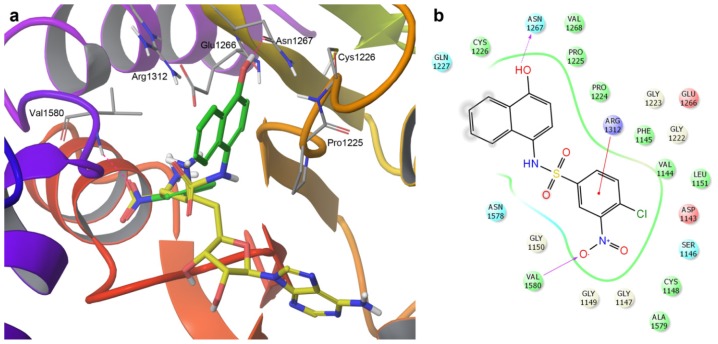
(**a**) Induced-fit docking pose of **SW155246** (carbon atoms in green) with human DNMT1. The co-crystal sinefungin (carbon atoms in yellow) is shown for reference; (**b**) Two-dimensional interaction diagram of the binding model of **SW155246**. Acidic, hydrophobic, basic, polar, and other residues at the active site are represented by red, green, purple, blue, and gray spheres, respectively. Hydrogen bonds between the ligand and backbone or side chains are shown in dashed pink lines. The π-cation interaction is shown with a red line.

Figure 4 shows three- and two-dimensional representations of the predicted binding mode obtained with IFD for **SW155246**. The structure of the co-crystal sinefungin that binds in the co-factor binding site is shown in Figure 4a for reference. Figure S1 in the Supporting Information shows a comparison of the predicted binding mode of the active compound **SW155246** and their structural analogues **SW155246-1** and **SW155246-2**. A major difference between the predicted binding modes for the three sulfonamides is that the active compound **SW155246** occupies part of the co-factor binding site as well as the catalytic site (Figure S1). The hydroxyl group of **SW155246** makes a distinct hydrogen bond interaction with the side chain of Asn1267 in the ENV motif of the catalytic site [19]. Of note, Ans1267 is adjacent to the amino acid Glu1266 that participates directly in the mechanism of cytosine C5 methylation by stabilizing the substrate [19]. An additional hydrogen bond interaction was observed between the nitro group of **SW155246** and the backbone of Val1580. A π-cation interaction is predicted with Arg1312 in the RXR motif. Arg1312 participate in the mechanism of cytosine-C5 methylation [20]. Interactions between Arg1312 and several other DNMT1 inhibitors, including 5-azacytidine (Figure 1) have been reported [16,20]. In contrast, the two inactive compounds are predicted to occupy only the binding site of the co-factor (Figure S1).

In addition to the different binding energies obtained with regular and IFD for the three sulfonamide compounds (Table 1), the binding poses obtained with regular docking were considerable different from the poses obtained with IFD (Figure S2 in the Supporting Information). In particular the orientation in the binding pocket of compounds **SW155246** and the methoxy analogue were significantly different. Of note, in the binding orientation obtained with regular docking, the active compound does not occupy part of the catalytic site (Figure S2). Residues that had the largest mobility after IFD were Arg1574, Gln1227 and Arg1312 in the docking with **SW155246**, and also Asn1578 in the docking with **SW155246-1** and **SW155246-2** (data not shown). These results further emphasize the importance of IFD to generate binding poses that are consistent with the observed experimental activity.

**SW155246** and their structural analogues are examples of three-dimensional activity cliffs, e.g., compounds with very similar structure but different binding mode [21]. For the series of compounds studies in this work, while the hydroxyl-containing molecule is active, methylation of loss of the OH group abolish activity. The substantial different binding mode predicted with IFD may explain, at least in part, the very different biological activity. We have used docking to interpret activity cliffs for other targets [22].

## 3. Experimental Section

The protein structure of human DNMT1 bound to sinefungin (PDB ID: 3SWR) was prepared using the Protein Preparation Wizard [23] implemented in Maestro [24] with the following steps: (i) Water molecules and all ligand atoms except sinefungin were removed; (ii) Hydrogen atoms were added; (iii) Protonation states of entire systems were adjusted to the pH range of 7.0 +/− 3.0 using Epik; (iv) Hydrogen bond networks and flip orientations/tautomeric states of Gln, Asn, and His residues were optimized; (v) The geometry optimization was performed to a maximum RMSD of 0.1 Å with the OPLS2005 force field. The chemical structures of **SW155246** and its analogues were built using Molecular Operating Environment (MOE), version 2013.08 [25]. The chemical structure of sinefungin was extracted from the crystallographic structure; hydrogen atoms were added and the most favorable protonation state was calculated with Protein Preparation Wizard.

For flexible docking, the grid box was centered on the coordinates of sinefugin to include the cofactor and substrate binding sites (setting the option dock ligands with length of 21 Å—the inner box had a size of 10 Å and the outer box a size of 31 Å) Docking was conducted using Glide XP (extra precision) [26]. Docked poses with the best (lowest) Glide XP score were selected for analysis.

For IFD, we employed the IFD protocol implemented in the Schrödinger software suite [27–29]. The ligands in Figure 2 were docked into the crystal structure of human DNMT1 using the following steps: (i) The receptor grid was defined as an enclosing box at the centroid of the top ranked conformation of **SW155246** using regular docking using an automated-generated box size by the IFD protocol; (ii) In the initial Glide docking stage, a soften potential docking with van der Waals radii scaling of 0.5 for the protein and ligand was performed retaining a maximum number of 20 poses per ligand; (iii) Residues within 5.0 Å of ligand poses were kept free to move in the Prime refinement step, and the side chains were further optimized; (iv) Poses within 30 kcal/mol of the lowest energy structure obtained in the previous step were re-docked using Glide XP; (v) The binding energy (IFDScore) for each output pose was computed as implemented in the IFD protocol. This score is the sum of the GlideScore from the redocking step and 5% of the Prime energy from the refinement calculation [27]. The most favorable binding conformations of each ligand complex, as measured by the IFDScore, were selected for analysis.

## 4. Conclusions

In this Communication, we report the binding model of **SW155246** with human DNMT1. Using IFD we provide a structural basis of the large difference observed in activity of this distinct sulfonamide inhibitor with closely related structural analogues obtained from HTS. We concluded that IFD had a significant impact on the docking scores and the predicted binding modes as compared to docking where the structure of the protein is kept fixed. Notably, the active compound **SW155246** showed a significantly different binding mode as compared to the structural analogues. We concluded that **SW155246** occupy part of the co-factor binding site as well as the catalytic site making a distinct hydrogen bond interaction with Asn1267. IFD was employed to account for protein flexibility and helped to provide a structural explanation of three-dimensional activity cliffs. Other approaches that could be used in future work for modeling protein flexibility is to conduct docking with multiple conformations of human DNMT1 obtained through crystallographic information or molecular dynamics. Understanding the SAR, in particular subtle changes that have a large impact on the activity, has a major importance to optimize the activity of novel inhibitors [30]. This work also illustrates the synergy between computational studies and reported experimental screening to further advance the knowledge of protein–ligand interactions relevant to a promising epigenetic target.

## Supplementary Information



## Figures and Tables

**Table 1.** Docking scores as computed with Glide XP using flexible docking and induced-fit docking (IFD). The IFD scores are also reported.

Compound	Flexible docking	IFD	

	Glide XP	Glide XP	IFDScore
**SW155246**	−4.817	−5.858	−1009.528
**SW155246-1**	−4.312	−5.244	−1008.860
**SW155246-2**	−4.370	−5.819	−1009.967
