# Supplementary material for: Rationalization of Activity Cliffs of a Sulfonamide Inhibitor of DNA Methyltransferases with Induced-Fit Docking
Source: Int J Mol Sci. 2014 Feb 21;15(2):3253–61. doi: 10.3390/ijms15023253 (PMC3958909; doi:10.3390/ijms15023253)
Supplement: Supplementary file 1 [file ijms-15-03253-s001.pdf]

Supplementary Information

Figure S1. Comparison of the induced-fit poses predicted for **SW155246** (carbon atoms in green), **SW155246-1** (carbon atoms in pink) and **SW155246-2** (carbon atoms in light blue). The co-crystal sinefungin (carbon atoms in yellow) is shown for reference. Hydrogen bonds between **SW155246** and the protein are shown in dashed pink lines.

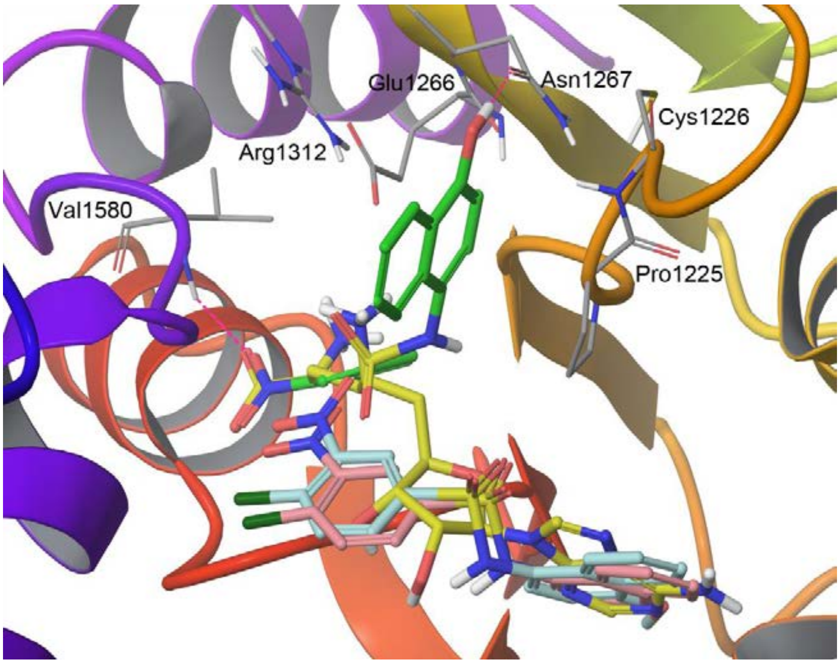

Figure S2. Predicted binding mode for **SW155246** with induced-fit docking (carbon atoms in light green) and regular docking (carbon atoms in dark green).

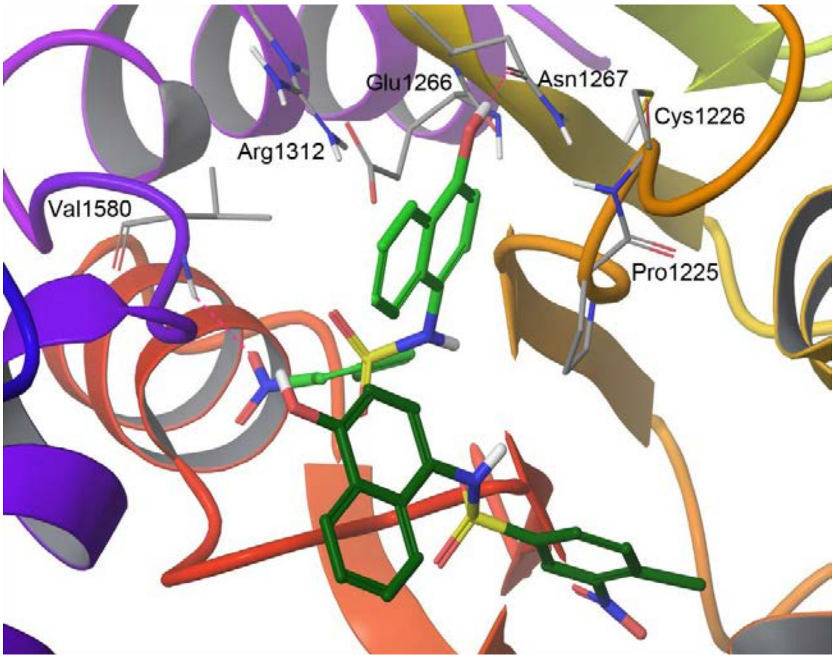

© 2014 by the authors; licensee MDPI, Basel, Switzerland. This article is an open access article distributed under the terms and conditions of the Creative Commons Attribution license (http://creativecommons.org/licenses/by/3.0/).
